# Association of body image perception and (dis)satisfaction with adiposity in adults: The Pró-Saúde study

**DOI:** 10.1371/journal.pone.0304987

**Published:** 2024-06-10

**Authors:** Magno C. Cabral, Gabriela M. O. Coelho, Natalia Oliveira, Daniela S. Canella, Raiane L. O. Brasil, Tatiana A. M. Campos, Eduardo Faerstein, Flávia F. Bezerra

**Affiliations:** 1 PostGraduate Program of Food, Nutrition and Health, Rio de Janeiro State University, Rio de Janeiro, Brazil; 2 Institute of Nutrition, Rio de Janeiro State University, Rio de Janeiro, Brazil; 3 Institute of Social Medicine, Rio de Janeiro State University, Rio de Janeiro, Brazil; Instituto Nacional de Cardiologia Ignacio Chavez, MEXICO

## Abstract

**Introduction:**

Body image distortion and/or dissatisfaction may occur primarily due to body fat accumulation and/or distribution. The aim of this study was to evaluate the frequency of body image perception and (dis)satisfaction categories in adult men and women according to the adiposity classification.

**Methods:**

This is a cross-sectional study (n = 514; 33–79 years; 265 women) that is part of a prospective cohort (Pró-Saúde study). Adiposity measurements were determined by two methods: anthropometry, used to calculate the body mass index (BMI) and dual-energy X-ray absorptiometry (DXA), to estimate body fat percentage. Participants were grouped as “no excess adiposity” and “excess adiposity”, considering BMI and body fat percentage (>30% for men, >40% for women). Perception and (dis)satisfaction with body image were evaluated using the Kakeshita scale, composed by 15 silhouettes, developed for the Brazilian population. Degree of distortion (perceived BMI ‐ real BMI) and dissatisfaction (perceived BMI ‐ desired BMI) were calculated.

**Results:**

A high proportion of men (58.6%; 74.3%), and especially of women (82.6%; 86.8%), presented body size overestimation and dissatisfaction due to excess weight, respectively. A relevant fraction of the women (32.6%) and men (30.8%) who were dissatisfied due to excess weight did not present excess adiposity, especially if classified by DXA. Variability in degree of distortion was hardly explained by anthropometric and DXA variables in women (<5%) and men (∼22%). Both anthropometric and DXA measurements accounted for ∼30% and ∼50% of the variability in degree of dissatisfaction among women and men, respectively.

**Conclusion:**

Our results suggest a high frequency of body image distortion in Brazilian adult individuals, as well as dissatisfaction with excess weight, especially among women with excess adiposity. The findings indicate that anthropometric measurements explain much of the variability in degree of body image dissatisfaction in men, with no apparent advantage of the use of more refined DXA measurements.

## Introduction

Body image is a multidimensional concept that involves individual`s perceptions, thoughts, feelings, and attitudes about their own body [1]. The misperception, or distortion of body size, can lead to negative health implications, such as weight gain and increased adiposity [2–4]. Similarly, there is evidence that body image dissatisfaction can be associated with health damage, e.g., adoption of unhealthy weight control behaviors, including prolonged fasting, use of food substitutes (i.e., powder or a special drink), diet pills, and laxative or diuretic use [5, 6]. Figure rating scales with visual representations of body silhouettes are among the most used methods to assess a distorted self-perception of body dimensions as well as the dissatisfaction with body image [7]. Given that the performance of scales is influenced by demographic and cultural characteristics of a given population, the use of scales developed or culturally adapted for the population under study is highly recommended [7]. Kakeshita and colleagues developed and validated a silhouette scale for Brazilian adults and elderly individuals, encompassing a wide range of BMI in 15 figures, thus providing a comprehensive representation of the population [8, 9].

Studies evaluating body image distortion and dissatisfaction in middle-aged adults gained greater attention from the scientific community in the last decade [10–16]. People’s distorted perception of their own body has been found to occur in a large part of the adult population, especially women, for whom frequencies of body size overestimation may reach 70–80% [15, 16]. Similarly, women also presented a high prevalence (60 to 70%) of body image dissatisfaction due to excess weight [11, 15, 17]. Body size overestimation [17, 18] and underestimation [10, 19] have been associated with increasing BMI. Body image dissatisfaction due to excess weight also seems to be frequently associated with a higher BMI [17, 18, 20]. A higher degree of body image misperception and dissatisfaction in individuals with overweight and/or obesity may impair their awareness of the need for obesity-related health treatment and may also encourage unhealthy eating behaviors [3, 5]. This may be particularly relevant given the tendency towards an increase in the prevalence of overweight/ obesity worldwide [21], including Brazil, where the prevalence of these combined conditions approaches 70% [22].

In addition to BMI, which is limited in accurately reflecting adiposity, there is evidence that anthropometric measures can contribute to explain body image distortion or dissatisfaction [13, 17], which gives the idea that the body fat accumulation, or its distribution, may be of particular relevance for body image evaluation. Consistently, body fat (estimated using skinfold thickness) predicted the degree of body dissatisfaction of young adult women [13], and waist circumference importantly explained body image (dis)satisfaction in older women [17]. Therefore, in the present study, we raised the hypothesis that the more accurate measure of body fat and distribution using dual-energy X-ray absorptiometry (DXA) would contribute to explain the variability in body image distortion and dissatisfaction. Better understanding body image perception and (dis)satisfaction according to adiposity severity may assist professionals in refining the design of weight-control strategies and addressing other healthcare issues. This, in turn, can contribute to combating the obesity epidemic and its associated health implications. The aim of the study was to evaluate the frequency of body image perception and (dis)satisfaction categories in adult women and men according to the adiposity classification. We also compared these frequencies using adiposity classifications derived from BMI and DXA body fat measurements. Additionally, we evaluated the association between degrees of body image distortion/dissatisfaction and different body fat measurements.

## Materials and methods

### Study design and population

This is a cross-sectional analysis of the Pró-Saúde Cohort study (State University of Rio de Janeiro- Brazil) aiming to investigate socioeconomic and psychosocial aspects associated with health conditions and behaviors [23]. During the fourth phase of the study (2011–2013), a subsample of 520 individuals ‐ constituting approximately 20% of the baseline cohort according to the proportion of sex, age, and schooling strata ‐ was invited to undergo complementary assessments. These assessments included the application of a sociodemographic questionnaire ‐ including data on menopausal status and on medication use ‐ a body image self-assessment, an anthropometric measurement, and a body composition assessment by dual-energy X-ray absorptiometry (DXA). Four participants who did not complete the DXA assessment and two who did not perform the body image self-evaluation were excluded from the present study (totalizing five women from 46 to 59 years and from 21.8 to 28.9kg/m^2^; and one men aged 48 years and 29.7 kg/m^2^). Thus, 514 participants were included in the analyses. The study was registered in the National Ethics in Research System (SISNEP), submitted for analysis and approved by the Ethics Council of the Institute of Social Medicine of the State University of Rio de Janeiro (CAAE: 04452412.0.0000.5260). The project was carried out after the written consent of the volunteers, who were previously informed of the purpose of the study.

### Anthropometry and DXA measurements

Total body mass was determined by using a digital scale (Filizola) with a 0.1 kg precision. Height was measured by using a fixed stadiometer (SECA) with a 0.1 cm precision. Based on these data, the body mass index (BMI) was calculated, and the study participants were classified according to the criteria defined by the World Health Organization (WHO) (1998). Flexible and non-extensible tape measure was used to measure waist and hip circumference.

The body composition of the study participants was evaluated by DXA (Lunar iDXA, GE-Lunar, Milwaukee, WI, USA). Data on total fat mass (kg) and android and gynoid regions (kg) were collected using the enCore2008 software version 12.20. The Corescan VAT software was used to collect data on visceral adipose tissue. Body fat percentage (percentage of total body fat relative to total body mass) was estimated, and men and women with a body fat percentage above 30% and 40%, respectively, were classified with obesity [24–26]. The DXA scans were all performed by the same evaluator and the equipment was calibrated according to the protocol established by the manufacturer. Details of the testing protocol and equipment calibration were previously described [27]. All the anthropometric and DXA measurements were performed at the Interdisciplinary Laboratory for Nutritional Evaluation at the Institute of Nutrition/ UERJ.

For the purposes of matching the classification of BMI-based adiposity measurements and body fat percentage, the participants were grouped as “no excess adiposity” and “excess adiposity”. The term no excess adiposity was applied to the participants presenting underweight and normal weight, when assessed by BMI, and to the participants without obesity when assessed by body fat percentage (DXA). The term “excess adiposity” was applied to subjects with overweight and obesity when assessed by BMI, and to participants classified with obesity, according to body fat percentage by DXA.

### Body image self-assessment

Body image assessment was performed by applying a silhouette rating scale designed and validated for the adult Brazilian population [8, 9]. The scales contain 15 body images for each sex corresponding to mean BMI ranging from 12.5 kg/m² to 47.5 kg/m², with images differing by 2.5 kg/m². Therefore, it is assumed that there is a variation of 1.25 kg/m² from the mean values for each image. Details of the use of information collected by applying the scale in this population were previously published [28]. In summary, after body images were presented, participants were asked to indicate which silhouette most closely represented their body size on that day (perceived silhouette) and which silhouette best represented the body they wanted to have (desired silhouette). The corresponding number of the silhouette (ranging from 1 to 15) chosen by individuals was recorded. The real silhouette was determined by converting the real BMI value (measured through anthropometric assessment) to the silhouette rating scale, while considering the variations of 1.25kg/m².

Body image perception was assessed by the difference between the perceived silhouette and the real silhouette (perceived ‐ real). Participants were then categorized as previously described [9, 28]. Briefly, negative values indicated body size underestimation while positive values indicated body size overestimation. Values equal to zero indicated no body image distortion. The assessment of the participants’ body image (dis)satisfaction was based on the difference between the perceived silhouette and the desired silhouette (perceived ‐ desired). Negative values indicated body image dissatisfaction due to thinness while positive values suggested body image dissatisfaction due to excess weight. Values equal to zero indicated body image satisfaction [9, 11, 28]. Additionally, absolute values obtained from the differences between the perceived BMI and the real BMI, as well as between the perceived BMI and the desired BMI, were used to estimate the degree of distortion and dissatisfaction, respectively, on a continuous scale.

### Statistical analysis

The variables were described by following classic procedures (frequencies, means, and standard deviation). Differences between women and men were evaluated by the chi-square test for categorical variables and by Student’s t-test for continuous variables. In order to additionally explore the physiological phenomena of aging, we conducted subgroup analysis in which women were categorized according to menopausal status (menopause: permanent cessation of menses for a consecutive period of 12 months), and men categorized by age (< 50 years and ≥ 50 years). Differences between these subgroups were also assessed using the chi-square test and Student’s t-test. The frequency of different perception and (dis)satisfaction categories, as well as degree of body image distortion and dissatisfaction, were described based on the presence or absence of excess adiposity. Associations between degree of distortion and dissatisfaction and the adiposity measurements, determined by anthropometry and DXA, were investigated by using multiple linear regression analysis. Associations with each variable were evaluated separately (crude model) and after mutual adjustment for age and for the variables of each “set” (anthropometry or DXA, adjusted model). All assumptions of the linear models have been tested, and multicollinearity was diagnosed using a variance inflation factor (VIF) > 5. P values<0.05 were considered significant. Statistical analyses were performed using the SPSS software version 17.0 (SPSS, Inc.).

## Results

### General characteristics of the population

The study population consisted of 265 women and 249 men. Table 1 shows sociodemographic, anthropometric, adiposity, and body image characteristics. The population ranged between 33 and 79 years old; the mean age was 52.1 ± 8.0 years for women and 51.2 ± 7.9 years for men. The participants predominantly self-declared as white (∼48%), had college education or higher (∼54%), and received less than 3 minimum wages (∼46%). Women exhibited higher measurement values for hip circumference, total fat mass, body fat percentage, and gynoid fat mass, while men had greater measurement values for waist circumference, waist/hip ratio, and visceral adipose tissue. There were no differences in android fat mass between men and women. According to BMI, most participants presented with excess weight (overweight ∼41% or obesity ∼30% for both men and women). According to body fat percentage, most women (60%) and men (55%) presented with obesity (Table 1). Participants identified themselves as users of antihypertensives (37%), antihyperglycemics (10%), and antihyperlipidemics (10.5%).

**Table 1:** Sociodemographic, anthropometric and DXA measures of adiposity and body image self-perception and (dis)satisfaction of the population. Pró-Saúde Study (2012–2013).

	Women (n = 265)	Men (n = 249)	
	Mean ± SD or n (%)	Mean ± SD or n (%)	P-value^1^
**Age (years)**	52.1 ± 8.0	51.2 ± 7.9	0.222
**Race/ Skin color** **³**			
Black	69 (26.3)	38 (15.5)	0.03
Mixed-race	74 (28.2)	72 (29.4)	
White	116 (44.3)	132 (53.9)	
Indigenous or Asian	3 (1.2)	3 (1.2)	
**Educational attainment** ^**4**^			
High school or less	120 (45.8)	111 (44.8)	0.813
College education or higher	142 (54.2)	137 (55.2)	
**Equivalent household income (per capita, minimum wages)** ^**5**^			
< 3 minimum wages	119 (45.3)	118 (47.6)	0.642
3–6 minimum wages	109 (41.4)	93 (37.5)	
> 6 minimum wages	35 (13.3)	37 (14.9)	
**Body mass (kg)**	71.9 ± 14.5	82.3 ± 15.3	<0.001
**Height (m)**	1.60 ± 0.6	1.72 ± 0.7	<0.001
**Waist circumference**	95.8 ± 12.4	98.7 ± 12.3	0.008
**Hip circumference**	106.0 ± 10.4	101.6 ± 8.2	<0.001
**Waist/Hip Ratio**	0.90 ± 0.07	0.97 ± 0.06	<0.001
**BMI (kg/m** ^**2**^ **)**	28.1 ± 5.4	27.6 ± 4.6	0.262
**BMI categories**			0.267
Underweight	3 (1.1)	2 (0.8)	
Normal weight	73 (27.5)	69 (27.7)	
Overweight	109 (41.1)	104 (41.8)	
Obesity	80 (30,3)	74 (29.7)	
**Total fat mass (kg)** ^**2**^	30.4 ± 9.9	25.6 ± 9.2	<0.001
**Body fat percentage** ^**2**^	41.7 ± 5.8	30.4 ± 6.4	<0.001
**Body fat percentage categories** ^**2**^			0.292
No obesity	107 (40.4)	112 (45.0)	
With obesity	158 (59.6)	137 (55.0)	
**Visceral adipose tissue (kg)** ^**2**^	1.0 ± 0.6	1.6 ± 1.0	<0.001
**Android fat mass (kg)** ^**2**^	2.6 ± 1.1	2.7 ± 1.2	0.654
**Gynoid fat mass (kg)** ^**2**^	5.6 ± 0.2	3.8 ± 1.4	<0.001
**Body image perception categories**			<0.001
Underestimation of body size	21 (7.9)	65 (26.1)	
No distortion	25 (9.4)	38 (15.3)	
Overestimation of body size	219 (82.6)	146 (58.6)	
**Degree of body image distortion**	5.3 ± 4.6	2.5 ± 4.8	<0.001
**Body image (dis)satisfaction categories**			0.001
Dissatisfied due to thinness	17 (6.4)	26 (10.4)	
Satisfied with body size	18 (6.8)	38 (15.3)	
Dissatisfied due to excess weight	230 (86.8)	185 (74.3)	
**Degree of body image dissatisfaction**	7.2 ± 5.7	5.6 ± 5.9	<0.001

^1^P-values determined by T-test for independent samples (continuous variables) or Chi-square test (categorical variables).

^2^Data collected using dual-energy x-ray absorptiometry (DXA).

^3^Due to missing data, women n = 262 and men n = 245

^4^Due to missing data, women n = 262 and men n = 248

^5^Due to missing data, women n = 263 and men n = 248

### Frequency of body image perception and (dis)satisfaction

The distribution in body image perception categories was different in men and women (Chi-square, P<0,001). More than 80% of women overestimated their own body size. Although overestimation was present in the majority of men (∼60%), underestimation of their own body size was also frequent (∼25%). There was also a difference between men and women in the distribution of body image (dis)satisfaction categories (P<0,001). More than 85% of women and 74% of men were dissatisfied with their body image due to excess weight. Only 7% of women and 15% of men were satisfied with their body image (Table 1). There were no significant differences in the distribution of body image perception and (dis)satisfaction categories according to the use of antihypertensives, antihyperglycemics, and antihyperlipidemics medications. However, there was a trend towards (Chi square, P = 0.08) a higher frequency of individuals dissatisfied due to excess weight among the users of antihypertensives drugs (data not shown).

Additional subgroup analysis to explore aging resulted in no significant difference in the distribution of perception and (dis)satisfaction categories between men under and over 50 years of age (Chi-square, P = 0.14 and P = 0.12, respectively). However, the perception of body image differed between menopausal (n = 166) and non-menopausal women (n = 99; Chi-square, P = 0.046). Among menopausal women, 86.1% (vs. 76% of non-menopausal women) overestimated their body image. There was no significant difference between the subgroups according to age (men) or menopausal status (women) in the degree of distortion (P = 0.54 for age; P = 0.21 for menopausal status) and dissatisfaction (P = 0.08 for age; P = 0.06 for menopausal status) (data not shown).

### Body image perception categories according to adiposity classification

Among women, only for those who overestimated their body size, there was a difference in the frequency of distribution between the groups: “no excess adiposity” and “excess adiposity” depending on the method used (BMI *vs*. body fat percentage; P<0.001; Table 2). The frequency of women that did not present with body image distortion was similar in both groups: “no excess adiposity” and “excess adiposity”, and there was no difference between methods. Among men, the use of different classification methods resulted in different frequencies of participants with and without excess weight for all categories of body size perception. Among men who did not present body image distortion, most had excess adiposity when assessed by BMI, while most did not present with excess adiposity according to body fat percentage (Table 2). It is worth pointing out that, for both men and women, most participants with excess adiposity (regardless of the classification method) were in the body size overestimation category.

**Table 2:** Frequency of body image perception categories according to the adiposity classification using BMI and body fat percentage. Pró-Saúde Study (2012–2013).

	Women			Men		
	Underestimation of body size^1^	No distortion	Overestimation of body size	Underestimation of body size	No distortion	Overestimation of body size
**According to BMI**						
No excess adiposity	14 (66.7)	11 (44.0)	51 (23.3)	39 (60.0)	12 (31.6)	20 (13.7)
Excess adiposity	7 (33.3)	14 (56.0)	168 (76.7)	26 (40.0)	26 (68.4)	126 (86.3)
**According to body fat %** ^**2**^						
No excess adiposity	15 (71.4)	12 (48.0)	80 (36.5)	50 (76.9)	21 (55.3)	41 (28.1)
Excess adiposity	6 (28.6)	13 (52.0)	139 (63.5)	15 (23.1)	17 (44.7)	105 (71.9)
P-value^3^	0.629	0.689	<0.001	0.001	0.003	<0.001

^1^Values indicate n (%) for categorical variables.

^2^ Body fat percentage was determined by dual-energy x-ray absorptiometry (DXA).

^3^ Chi-square p-values for the comparison between methods for adiposity classification within categories of body image perception.

### Body image (dis)satisfaction categories according to adiposity classification

The two methods used for evaluating excess adiposity also resulted in differences in the distribution of categories with and without excess adiposity depending on body (dis)satisfaction category in men and women (Table 3). Most of the individuals who were dissatisfied due to excess weight presented with excess adiposity, and the frequency was higher when the BMI method was used, both in women (80.9% *vs* 67.4%) and in men (88.6% *vs* 69.2%). Among men who were satisfied with body image, there was a high frequency of individuals in the group with no excess adiposity, especially when classified according to body fat percentage (81.6%) (Table 3).

**Table 3:** Frequency of body image (dis)satisfaction according to the adiposity classification using BMI and body fat percentage. Pró-Saúde Study (2012–2013).

	Women			Men		
	Dissatisfied due to thinness^1^	Satisfied	Dissatisfied due to excess weight	Dissatisfied due to thinness	Satisfied	Dissatisfied due to excess weight
**According to BMI**						
No excess adiposity	15 (88.2)	17 (94.4)	44 (19.1)	24 (92.3)	26 (68.4)	21 (11,4)
Excess adiposity	2 (11.8)	1 (5.6)	186 (80.9)	2 (7.7)	12 (31.6)	164 (88,6)
**According to body fat %** ^**2**^						
No excess adiposity	15 (88.2)	17 (94.4)	75 (32.6)	24 (92.3)	31 (81.6)	57 (30,8)
Excess adiposity	2 (11.8)	1 (5.6)	155 (67.4)	2 (7.7)	7 (18.4)	128 (69,2)
P-value^3^	1.000	1.000	<0.001	1.000	0.036	<0,001

^1^Values indicate n (%) for categorical variables.

^2^ Body fat percentage was determined by dual-energy x-ray absorptiometry (DXA).

^3^ Chi-square p-values for the comparison between methods for adiposity classification within categories of body image (dis)satisfaction.

### Adiposity measures association with degrees of body image distortion

Table 4 shows the associations between degree of distortion (perceived BMI ‐ real BMI), degree of dissatisfaction (perceived BMI ‐ desired BMI), and adiposity measurements (anthropometry or DXA). No association was found between age and the degree of distortion (P = 0.89 for men and P = 0.572 for women) and dissatisfaction (P = 0.12 for men and P = 0.08 for women) in both sexes. In both women and men, after mutual adjustment among anthropometric variables, degree of distortion presented an inverse association with total body mass, and a direct association with waist circumference (Table 4). We further analyzed the anthropometric model for women after excluding total body mass (due to VIF∼10) and observed no substantial modifications (β = 0.070 95%CI 0.010 to 0.148 for waist circumference, β = -0.022 95%CI -0.115 to 0.071 for hip circumference, r² = 0.022). In both men and women, after adjustment by the other variables of the DXA model, degree of distortion presented a direct association only with body fat percentage. Among women, the variables of the anthropometric measurement model and the DXA measurement model explained, respectively, about 5% and 2% of the variability in degree of distortion. Among men, both models explained about 22% of the variability (Table 4).

**Table 4:** Association between degrees of body image distortion and dissatisfaction and adiposity measures in women and men. Pró-Saúde Study (2012–2013).

	Degree of body image distortion^1^				Degree of body image dissatisfaction			
	Women^2^		Men		Women		Men	
	Crude model	Adjusted model^3^	Crude model	Adjusted model	Crude model	Adjusted model	Crude model	Adjusted model
**Anthropometric model**								
Total body mass	0.021	**-0.186**	**0.096**	**-0.141**	**0.205**	-0.070	**0.248**	**-0.135**
	(-0.018; 0.060)	**(-0.308; -0.063)**	**(0.058; 0.133)**	**(-0.243;-0.038)**	**(0.164; 0.246)**	(-0.198; 0.058)	**(0.211; 0.286)**	**(-0.232; -0.038)**
Waist circumference	**0.055**	**0.173**	**0.168**	**0.369**	**0.258**	**0.208**	**0.349**	**0.369**
	**(0.010; 0.100)**	**(0.066; 0.281)**	**(0.125; 0.212)**	**(0.257; 0.480)**	**(0.211; 0.304)**	**(0.095; 0.320)**	**(0.308; 0.390)**	**(0.263; 0.474)**
Hip circumference	0.044	0.117	**0.181**	-0.075	**0.284**	**0.184**	**0.488**	**0.220**
	(-0.009; 0.098)	(-0.00; 0.237)	**(0.112; 0.251)**	(-0.246; 0.096)	**(0.227; 0.341)**	**(0.057; 0.310)**	**(0.422; 0.555)**	**(0.058; 0.383)**
Adjusted R^2^		0.053		0.223		0.325		0.545
**DXA model**								
Body fat %	**0.111**	**0.265**	**0.351**	**0.413**	**0.526**	**0.352**	**0.641**	**0.351**
	**(0.016; 0.207)**	**(0.074; 0.455)**	**(0.268; 0.434)**	**(0.220; 0.605)**	**(0.426; 0.626)**	**(0.153; 0.551)**	**(0.558; 0.724)**	**(0.161; 0.541)**
Android fat mass	0.288	-0.406	**1.581**	0.356	**2.615**	**1.113**	**3.245**	**1.310**
	(-0.212; 0.787)	(-1.411; 0.599)	**(1.144; 2.018)**	(-0.716; 1.428)	**(2.086; 3.145)**	**(0.062; 2.165)**	**(2.815; 3.676)**	**(0.282; 2.390)**
Gynoid fat mass	0.110	-0.357	**1.198**	-0.646	**1.389**	-0.038	**2.682**	0.318
	(-0.201; 0.421)	(-0.919; 0.206)	**(0.806; 1.591)**	(-1.466; 0.175)	**(1.045; 1.734)**	(-0.627; 0.550)	**(2.287; 3.078)**	(-0.492; 1.127)
Adjusted R^2^		0.019		0.216		0.292		0.499

^1^Degree of body image distortion assessed as perceived BMI ‐ real BMI and degree of dissatisfaction assessed as perceived BMI ‐ desired BMI.

^2^All values are β (confidence interval).

^3^Each variable is adjusted for age and for the other anthropometric or DXA measures.

### Adiposity measures association with degrees of body image dissatisfaction

Degree of dissatisfaction was strongly associated with adiposity measures, both anthropometry and DXA, in both men and women. Among women, in the adjusted model of anthropometric measurements, degree of dissatisfaction was directly associated with waist and hip circumferences. The anthropometric model for women was also conducted after excluding total body mass (due to VIF∼10) and resulted in no substantial modifications (β = 0.174 95%CI 0.093 to 0.255 for waist circumference, β = 0.118 95%CI 0.023 to 0.213 for hip circumference, r² = 0.329). Among men, degree of dissatisfaction was associated with all variables of the anthropometric measurement model. For both men and women, after adjustment for the other variables of the DXA model, degree of dissatisfaction presented a direct association with body fat percentage and android fat mass. Among women, the variables of the anthropometric measurement model and the DXA measurement model explained, respectively, about 33% and 29% of the variability in the degree of dissatisfaction. Among men, the models explained about 55% and 50%, respectively (Table 4).

## Discussion

In the present study, assessing Brazilian adults, we found that a high proportion of women and men distorted the perception of their body image; they mostly perceived their body as larger than its real size. Additionally, most participants were dissatisfied due to excess weight. The frequency of dissatisfaction was high even among participants without excess adiposity, regardless of the method (BMI or DXA body fat percentage) used for such classification. For men and women, both the distortion and the dissatisfaction degree were associated with adiposity measures. It is worth noting that, in the case of women, the set of measures (anthropometric or DXA-assessed) hardly explains the variability in the degree of distortion.

### Frequency of body image perception and (dis)satisfaction: The scale matters

Studies evaluating adult men and women of a wide age range (between 18 and 70 years old) and with a high prevalence of overweight and obesity (between 60 and 80%) found that the frequency of correct body image perception ranged from 39 to 55% [10, 29]. It should be noted that those studies used the figure rating scale proposed by Stunkard, Sorensen & Schulsinger (1983) [30] to evaluate African Americans [29] and Spanish [10] adults. Applying the figure rating scale proposed by Kakeshita [9] to Brazilian elderly individuals (>60 years, n = 3263) with a 40% prevalence of overweight and obesity, a lower frequency of correct body image perception was observed, ranging from 19 to 36% in women and men, respectively [15]. In another study, applying the Kakeshita’s figure rating scale (2009) to Brazilian women (67± 4.3 years) with a mean BMI of 29.6kg/m² (±5.2kg/m²), it was found that 38% of the sample had a correct body image perception [17]. It was expected that applying the same instrument to individuals of the same nationality would account for a similar frequency of individuals without distortion, even though the slight age range difference. However, in the present study, there was an even lower frequency of individuals with no body image distortion, both in men (15.3%) and women (9.1%). Given that body image is a complex construct, it may be that several other emotional, cultural and socioeconomic factors not evaluated in the present study may have contributed to this low frequency of people presenting a correct body image perception.

The literature recognizes that the assessment of body image perception may vary according to the study populations and the assessment instrument being used [7]. The figure rating scale proposed by Stunkard et al. (1983) has nine figures, which are six less than the scale proposed by Kakeshita (2009). As a successful answer, for both scales, depends on the correct choice of only one figure, there is a greater likelihood of correct answers when using the Stunkard et al. (1983) scale. A previous study, using Kakeshita’s scale, found that the increase in the ranges of answers that were considered correct resulted in considerably higher frequencies (31.6% for women and 52.8% for men) of correct body image perception in Brazilian adults [28].

### Factors affecting body image perception

It is well accepted that women distort their body image more frequently when compared to men in terms of body size overestimation [12, 18]. Moreover, women experiencing menopause may have their body image negatively impacted by typical menopausal symptoms and changes in body composition [31, 32]. This is consistent with the findings of the present study, which observed differences in body image perception between menopausal and non-menopausal women. It is also well accepted that body size overestimation increases in the presence of BMI-assessed excess weight [18] or excess body fat measured by skinfolds [12]. Consistent with the literature, we found a higher rate of body size overestimation among women (83% vs. 59% in men). For both men and women, overestimation was more frequently found among individuals with excess adiposity, especially when classified by BMI. This result suggests that body image perception is more closely associated to body size than to specific information on fat accumulation. In addition, it is important to emphasize that the figure rating scale used in the present study was designed according to the BMI distribution of the Brazilian population [9].

In addition to physical aspects, the perceptual dimension of body image is influenced by several factors that include emotional and socio-cultural aspects [14]. According to the complexity of this dimension, the adiposity measures explored in the present study (anthropometry or DXA) hardly explained the variability in the degree of distortion, especially among women.

### Factors affecting body image dissatisfaction

It has been reported that body image dissatisfaction caused by excess weight is frequently (from 59% to 82%) observed among women [11, 14–16]. Previous studies have suggested that the pattern of female beauty frequently observed in Western cultures [33, 34] is characterized by the search for thinness [14, 34] which, at least in part, can explain this finding. Conversely, body dissatisfaction for men may be more diverse than for women: it included a concern with musculature and body shape [35]. For example, in previous studies, dissatisfaction due to thinness among men ranged from 3.7% to 67%, and can be attributed to the desire for lean body mass gain and a muscular physical appearance [11, 14, 34]. In the present study, although the frequency of men satisfied with their body image was about twice as much as that of women, dissatisfaction due to excess weight was very high in both sexes (87% of women and 74% of men). The high prevalence of overweight (∼40%) and obesity (∼30%) found in the study population may have contributed to these results.

Recent studies have shown that those who were perceived to be overweight were less likely to be satisfied with their body image [18, 20]. In the present study, we found that most participants dissatisfied due to excess weight were the ones with excess adiposity when the assessment method was DXA (∼68%) and especially when they were assessed on the basis of BMI (∼85%). However, it is worth pointing out that approximately 30% of participants without excess adiposity, assessed by DXA, also presented with body image dissatisfaction due to excess weight. It’s important to mention that the DXA definition of excess adiposity (i.e. total body fat percent >30% for men and >40% for women) utilized in the present study is widely used, but not universally agreed upon, with the cut-off values still being a matter of debate in the literature [36].

In addition, our results suggest that central adiposity measurements evaluated by anthropometry (waist and hip circumferences) and DXA (android fat mass) can help explain the variability in the degree of body image dissatisfaction. Importantly, the model generated from the anthropometric measurements of adiposity could explain much of the variability in the degree of dissatisfaction, especially in men, without apparent advantage of the use of more refined DXA-assessed measures.

### Strengths and limitations

A strength of the present study is that it clarifies the scenario of body image (dis)satisfaction and perception in Brazilian adults, an age range often neglected in studies on this theme. The study was further strengthened by employing the highly accurate measures of adiposity provided by DXA, besides the most commonly used anthropometric data. Given that the perceptual dimension of body image is intricately constructed, drawing from unconscious memories and past experiences, one of the main limitations of our study is that we did not address emotional, cultural and socioeconomic factors that could be of great importance in understanding self-perception of body image. In addition, the sample included in this study is composed of university staff, whose specific demographic and socioeconomic characteristics may differentiate them from the general population. Therefore, generalizing the results to a wider population should be done with due caution.

## Conclusion

Our results suggest a high frequency of body image distortion in Brazilian adult individuals, as well as dissatisfaction due to excess weight, especially among women with excess adiposity. Since distortion and dissatisfaction with body image may be related to the development of psychosocial disorders, it is important that health professionals are aware of the high frequencies observed in our population and consider the possibility of including this tool in the individual’s global assessment. For both sexes, the difference in methods used for assessing excess adiposity (BMI vs. DXA) was more evident among individuals who overestimated their body size and who were dissatisfied with their excess weight. The results also suggest that there are no advantages in employing DXA (more accurate measure) over anthropometry (a double indirect method) to better explain the factors that determine the variability of body image, refuting our hypothesis. It is noteworthy, though, that a simpler and more economic method seems sufficiently robust to explain most of body image variability (eg. ∼55% of the variability in the degree of body image dissatisfaction in men), except for the perceptual dimension in women. Given that body image refers to the multifaceted psychological experience of embodiment that encompasses one’s body-related self-perceptions and self-attitudes, including thoughts, beliefs, feelings, and behaviors influenced by biological and social characteristics; future research should address the emotional and sociocultural dimensions.
